# Factors associated with time to first healthcare visit, diagnosis and treatment, and their impact on survival among breast cancer patients in Mali

**DOI:** 10.1371/journal.pone.0207928

**Published:** 2018-11-29

**Authors:** Kirstin Grosse Frie, Bakarou Kamaté, Cheick Boudagari Traoré, Madani Ly, Brahima Mallé, Bourama Coulibaly, Andreas Wienke, Eva Johanna Kantelhardt

**Affiliations:** 1 Institute for Medical Epidemiology, Biostatistics and Informatics, Martin-Luther-University Halle-Wittenberg, Halle (Saale), Germany; 2 Institut of Pathology, University Hospital Point G, Bamako, Mali; 3 Oncology Department, Hôpital Luxemburg, Bamako, Mali; 4 Department of Gynaecology, University Hospital Halle (Saale), Germany; Iranian Institute for Health Sciences Research, ISLAMIC REPUBLIC OF IRAN

## Abstract

**Objective:**

To analyse patient and healthcare system related factors influencing the time to first healthcare visit, diagnosis and treatment of breast cancer patients in sub-Saharan Africa and the impact on survival in order to advise on early detection strategies.

**Methods:**

A prospective hospital cohort study was conducted at the only pathology department in Mali, at the University Hospital in Bamako. All the female patients with a breast cancer diagnosis between January and April 2016 were interviewed with a structured questionnaire (*N* = 64) to gather information about breast symptom recognition and first healthcare visit. Information on beginning of treatment and survival were collected at 18-months follow-up. Simple Cox regression analyses were performed.

**Results:**

The median time to first healthcare visit was 4.8 months, from first healthcare visit to diagnosis was 0.9 months and for the patients who started treatment (*N* = 46) the time from diagnosis to treatment was 1.3 months. Knowledge of breast-self-examination and correct symptom interpretation increased the chance of an earlier healthcare visit. Prolonged time to diagnosis was found with shorter duration to first healthcare visit, for working women compared to housewives and for those living within Bamako. Living outside Bamako and smaller tumour size (T1/T2) prolonged time to treatment. Visit of a traditional healer and larger tumour size (T3/T4) shortened survival time, whereas time to first healthcare visit and subsequent time to diagnosis had no influence on survival.

**Conclusions:**

Down-staging strategies are only useful if the continuum of breast cancer care is warranted for the majority of patients.

## Introduction

Breast cancer is one of the most common cancers among women in sub-Saharan Africa in terms of prevalence and incidence [1]. While incidence rates for sub-Saharan Africa are far below the ones known from high-income countries, they are likely to rise continuously due to an increase in life expectancy, changes in reproductive patterns and lifestyles [2]. Breast cancer mortality rates are high in sub-Saharan Africa as almost one in every two breast cancer patients dies [1]. A 5-year survival rate of 13.6% for breast cancer patients in Mali (not age standardised) [3] and a 5-year age-standardised relative survival of 12% in Gambia have been reported [4]. However, those survival estimates are considered less reliable and probably ignore a significant number of patients not being registered and dying without a diagnosis and treatment [3].

Clinical factors such as stage at diagnosis were reported as important determinants of survival. A study from Ethiopia showed that age and stage at diagnosis significantly influenced the length of metastatic free survival [5]. According to a review with data from 83 studies from sub-Saharan Africa, a median proportion of 75% breast cancer patients was diagnosed with stage III or advanced stages [6] and, among these, 36 studies reported the length of time between first symptom recognition and diagnosis with an average median duration of 8 to 12 months in most of the studies. While no association was found between time to diagnosis and late stage breast cancers on the study level, the authors still reasoned that “most advanced stage cancers might be a result of delayed diagnosis” [6] and that the large time frame leaves space for improvements.

To understand why breast cancer patients get diagnosed late in sub-Saharan Africa, some quantitative studies have analysed the length of time between symptom recognition and first healthcare visit and/or diagnosis among breast cancer patients and analysed related factors, as summarized in recent reviews [7, 8]. Quantitative and qualitative studies have described barriers at the individual and healthcare level, such as low breast cancer awareness, use of traditional medicine, difficulties in healthcare access, mistrust in and complicated navigation through the healthcare system, wrong diagnoses and no or delayed referral after a first healthcare visit, as well as costs and financial burden [8–11]. While time delays to treatment have rarely been reported [7], some studies solely focusing on treatment described fear of mastectomy, unavailability of specialist services and high costs of diagnosis and treatment as barriers [12–14]. The entire patient pathway from symptom recognition to beginning of treatment has, to our knowledge, only been reported in two cross-sectional studies, in Morocco [15] and recently in South Africa [16], which could be due to most study designs not analyze the continuum of care for patients after receiving a breast cancer diagnosis.

This pilot study in Mali, Western Africa, is to our knowledge the first that analysesd the entire patient pathway from first symptom recognition to beginning of treatment, following-up patients at least 18 months after diagnosis. Our study considered patient, disease specific and healthcare system related factors [17, 18] and analysed their association with time to first healthcare visit, diagnosis and treatment and their impact on overall survival.

## Materials and methods

### Study setting

The study took place in Bamako, the capital of Mali, in Western Africa and was part of a larger project on breast cancer diagnosis in Mali [10]. Breast cancer diagnoses are routinely performed at the only pathology department in the country, at the University Hospital Point G, where this study took place. Fine needle aspiration is the standard diagnostic procedure; some patients received a diagnosis of histopathology after biopsy or surgical interventions. Immune histopathology was not performed. Chemotherapy, surgery and radiotherapy are available in hospitals and private clinics in Bamako. At the beginning of the project, an association of breast cancer patients was contacted to discuss the project and to obtain advice on important research topics. For this study, breast cancer patients were involved in the pilot testing of the questionnaire to improve the comfortability of the interviews for patients and to include important items from the patient’s point of view.

### Data collection

We used a convenient sample as our study has an explorative character. All the female patients who received a breast cancer diagnosis at the department of pathology of the University Hospital Point G in Bamako, Mali, between January 1st and April 30th 2016 were included in this study (*N* = 64). Depending on the type of diagnosis, patients were interviewed at the pathology department the day of the fine needle aspiration or biopsy, or when they collected the results after an excisional biopsy. All patients were interviewed personally with a standardised questionnaire by two female medical students who were familiar with the study and interview techniques.

Patients were informed about the study and asked to confirm their willingness to participate. Ethical clearance was obtained by permission from the local ethics committee of the Medical Faculty in Bamako, Mali, with permission given for either written or oral consent. The implementation of written consent in the pilot phase confused and alienated the majority of patients, who were mainly illiterate and unfamiliar with a signing praxis, so we decided to obtain consent orally. Patients were included if they clearly stated their willingness to participate in the study, including participation in the interview, use of medical records and possible phone contact for follow-up.

The questionnaire was designed to analyse the patient pathway from first symptom recognition to beginning of treatment. The questionnaire was developed in accordance with questionnaires used for similar studies in Mexico [19] and Morocco [15] and in relation to the model of pathway to treatment [20]. The questionnaire can be found as supporting information in the original French (S1 Questionnaire) and translated English version (S2 Questionnaire). Dates of symptom recognition, first healthcare visit, diagnosis and start of treatment were collected. A calendar technique [21] was used to help patients to recall dates of symptom recognition and first healthcare visit due to breast related symptoms. The obtained information was confirmed by medical records, when available. The questionnaire also included items to describe the events in more detail (e.g., type of symptom recognised, type and number of medical doctors consulted, type of healthcare services) and factors that could influence the time intervals between the measured time points (e.g., visited a traditional healer, no referral). The collected data was linked with sociodemographic and clinical data routinely collected at the pathology service. The questionnaire was pretested with the interviewers and four patients in personal interviews, lasting about one hour. The questionnaire was in French but, if needed, interviews were conducted in the local language, Bambara. In the pilot phase, the interviewers were trained to translate the meaning of the questions into Bambara. Minor changes helped to improve understanding and the fluency of interviews. The personal interviews lasted about 20 minutes and took place in a protected room at the pathology department.

All interviewed patients or their families were contacted by telephone for follow-up between 18 and 23 months after diagnosis to receive information about the beginning of treatment and eventual date of death. Medical records were additionally searched at the main hospitals, where breast cancer surgery and chemotherapy are available. No information on treatment and survival was gathered for 9 patients.

### Data analysis

Of the total 66 patients registered at the pathology department of University Hospital Point G, 64 consented to participate. Simple Cox regression analyses were performed to analyse the influence of sociodemographic factors (age, occupation, marital status, residence, health insurance), patient related factors (knowledge of breast self-examination, visit of a traditional healer, first symptom interpretation), healthcare system related factors (facility and type of medical doctor at the first healthcare visit, first diagnosis, facility and doctor of diagnosis) and pathological diagnosis (tumour size, lymph node involvement) on the lengths of three different time intervals and on survival. The three time intervals were the time to first healthcare visit (time between recognition of the first symptom and first healthcare visit), time to diagnosis (time from first healthcare visit to diagnosis) and time to treatment (time from diagnosis to start of treatment). Crude hazard ratios (HR), 95% confidence intervals (CI) and *p* values are reported and interpreted in an explorative sense.

For the variables with the largest effect on overall survival in the simple Cox regressions (tumour size T3/T4, traditional healer, and health insurance status), Kaplan-Meier survival curves and log-rank test were additionally performed to illustrate the findings.

## Results

In the analyses of time to first healthcare visit and diagnosis, 64 patients were included. At the end of the study, 19 to 23 months after diagnosis, information on treatment uptake was available for 86% (*N* = 55) and survival status for 84% (*N* = 54), of which 20 patients had died.

**Fig 1:**
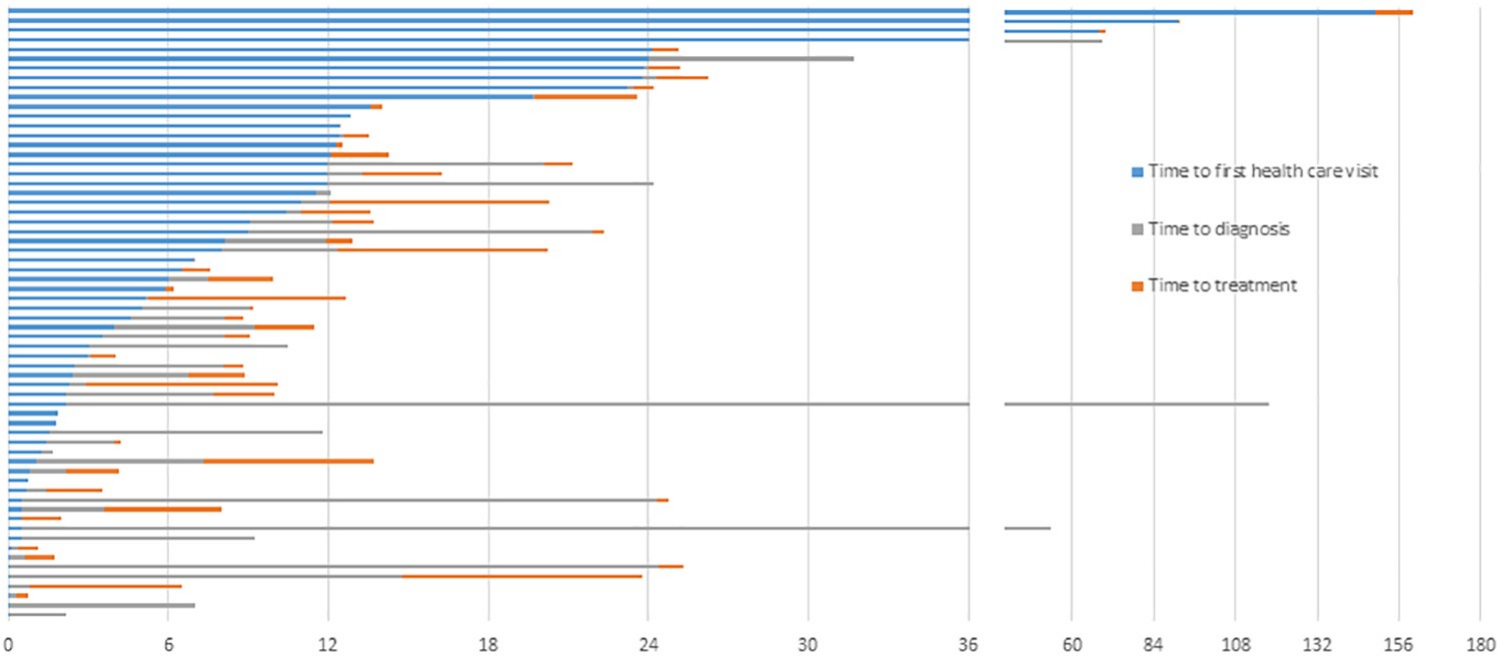
Distribution of the three consecutive time intervals in months (time to first healthcare visit, time to diagnosis and time to treatment) for each patient (*N* = 64).

The mean age of all patients was 45 years. The mean time to first healthcare visit was 11.6 months (range 0.3 to 149 months), with a median of 4.8 months. Mean time to diagnosis after first healthcare visit was 6.4 months (0 to 115.6 months), with a median of 0.9 months. For the 46 patients who started treatment, the mean time to treatment after diagnosis was 2.5 months (0.2 to 11 months), with a median of 1.3 months. The distribution of the three time intervals for each patient are shown in Fig 1. There are some outliers taking 2 years and longer to first healthcare visit, diagnosis or treatment.

Patients without knowledge of breast-self-examination or who interpreted the first symptoms as an infection or nothing serious had a higher risk of seeking healthcare later (Table 1). Furthermore, having no health insurance and being diagnosed with a T3 or T4 tumour was associated with an increased time to first healthcare visit. The hazard of having a longer consecutive time interval to a pathologically confirmed breast cancer diagnosis after a first healthcare visit was increased for working women compared to housewives, for women living in Bamako compared to women not residing in the capital of Mali and for married women compared to single, widowed or divorced women. With increasing age, the risk of having longer time intervals to diagnosis decreased. Women who first visited a community healthcare centre or a generalist and women who received no diagnosis or were diagnosed with an infection at the first healthcare visit had an increased time to diagnosis.

**Table 1:** Distribution, HR (95% CI) and *p* values for factors associated with time to first healthcare visit, diagnosis, treatment and survival.

	N	Time to first health care visit, N = 64		Time to pathological diagnosis, N = 64		Time to treatment begin, N = 46		Time to death, N = 45	
		crude HR (95% CI)	p	crude HR (95% CI)	p	crude HR (95% CI)	p	crude HR (95% CI)	p
**Age in 10 years**	64	0.98 (0.83;1.16)	0.81	1.2 (1.0; 1.5)	0.05	0.99 (0.81; 1.23)	0.94	1.04 (0.76; 1.41)	0.81
**Occupation**									
Housewife	34	1		1		1		1	
Working	30	1.3 (0.8; 2.2)	0.25	0.5 (0.3; 0.8)	0.01	0.9 (0.5; 1.6)	0.71	0.5 (0.2; 1.4)	0.17
**Married**									
Yes	45	1		1		1		1	
No	19	0.7 (0.4; 1.3)	0.26	1.5 (0.9; 2.7)	0.13	1.6 (0.9; 3.1)	0.14	1.3 (0.5; 3.2)	0.60
**Residence**									
Bamako	29	1		1		1		1	
Other	35	1.3 (0.8; 2.1)	0.38	1.9 (1.1; 3.2)	0.02	0.4 (0.2; 0.8)	0.01	0.6 (0.3; 1.5)	0.28
**Health Insurance**									
Yes	13	1		1		1		1	
No	51	0.6 (0.3; 1.2)	0.15	0.9 (0.5; 1.7)	0.83	2.0 (1.0; 4.2)	0.06	7.1 (0.9; 53.7)	0.06
**Knowledge of BSE**									
Yes	13	1		1				1	
No	51	0.3 (0.2; 0.6)	0.00	1.3 (0.7; 2.4)	0.4	1.3 (0.6; 2.9)	0.49	0.7 (0.3; 2.0)	0.53
**Traditional healer**									
Yes	23	1		1		1		1	
No	41	1.6 (0.9; 2.6)	0.09	1.1 (0.6; 1.8)	0.86	0.6 (0.3; 1.1)	0.11	0.3 (0.1; 0.8)	0.02
**Symptom Interpretation**									
Cancer	13	1							
Infection/ Nothing	51	0.4 (0.2; 0.8)	0.01						
**First Health care facility**									
Community	13	1		1					
Hospital	51	0.8 (0.5; 1.6)	0.58	1.5 (0.8; 2.8)	0.21				
**First medical doctor**									
Generalist	23	1		1					
Specialist	41	1.1 (0.6; 1.8)	0.82	1.5 (0.9; 2.5)	0.15				
**First Diagnosis**									
Cancer/Serious	38			1					
Infection/ Nothing	26			0.7 (0.4; 1.1)	0.15				
**Time to health visit (months)**	64			1.01 (1.00; 1.03)	0.04	1.003 (0.993; 1.013)	0.53	1.002 (0.987; 1.018)	0.77
**Time to Diagnosis (months)**	64					0.996 (0.962; 1.032)	0.84	0.98 (0.91; 1.05)	0.57
**Facility of diagnosis**									
University Hospital	48					1			
Other	16					0.8 (0.4; 1.5)	0.45		
**Doctor of diagnosis**									
Generalist/Gynecologist	29					1			
Oncologist/Surgeon	35					1.6 (0.9; 2.8)	0.16		
**Size of Tumor**									
T0/T1/T2	15	1		1		1		1	
T3/T4	49	0.6 (0.4; 1.2)	0.15	0.8 (0.5; 1.5)	0.55	4.1 (1.7; 10.1)	0.00	4.1 (1.7; 10.1)	0.00
**Lymph Node Involvement**									
N0	22	1		1		1		1	
N1/N2	42	1.4 (0.8; 2.4)	0.22	0.9 (0.5; 1.5)	0.62	0.9 (0.4;1.6)	0.61	2.9 (0.8; 9.8)	0.09

Every month increase in time to first healthcare visit, decreased the risk of prolonged time to diagnosis by 1%, while further analyses reveal that the total time to diagnosis (from first symptom recognition to diagnosis) is not longer for women with shorter times to first healthcare visit (Table 2).

**Table 2:** Median total time to diagnosis (from first symptom recognition to diagnosis) related to time to first healthcare visit in months.

Time to first health care visit (months)	N	Median total time to diagnosis (months)
≤ 3	28	3,4
3.1–6.0	7	8,1
6.1–12	13	12,1
≥12.1	16	23,7

Being married, residing outside Bamako compared to living in Bamako, having health insurance and visiting a traditional healer before the first healthcare visit increased the hazard of beginning treatment later. Women who were referred to the pathology service for diagnosis by an oncologist or surgeon and women with T3/T4 tumours started treatment earlier than women who were referred by a generalist or gynaecologist or with T1/T2 tumours, respectively.

The simple Cox survival analyses showed that being a housewife compared to women with working status, visiting a traditional healer before the first healthcare visit, larger tumour size (T3/T4) or lymph node involvement increased the hazard for death. Having no health insurance showed an increased hazard of HR = 7.1 (*p* = 0.06) of dying. The duration of time to healthcare visit and time to diagnosis had no influence on survival.

**Fig 2:**
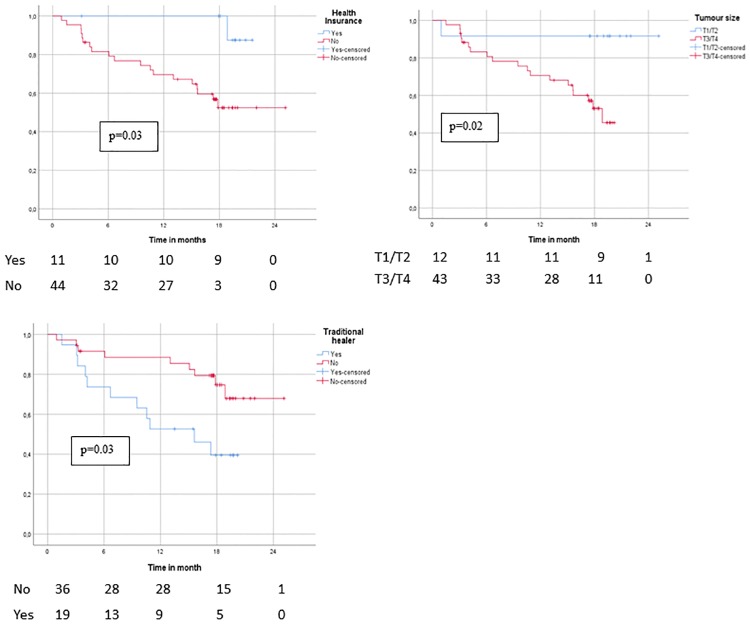
Kaplan-Meier overall survival curves and log-rank tests for health insurance status (yes/no), use of traditional healer (yes/no) and tumour size (T1/T2 and T3/T4).

The log-rank test and the Kaplan-Meier survival curves for the three variables with the largest effect on overall survival are presented in Fig 2. The results of the log-rank test indicate remarkable differences between the respective groups ((visit of a traditional healer (yes/no), tumour size (T1/T2 and T3/T4) and health insurance (yes/no)) on the overall survival rate.

## Discussion

### Principal findings

We presented time lengths to first healthcare visit, diagnosis and treatment start among Malian breast cancer patients and compared them with results from similar studies in sub-Saharan Africa published within the last 10 years (Table 3).

**Table 3:** Comparisons of time to first health care visit, diagnosis and treatment reported in studies from Africa.

Time Interval	Study	Country	Time in month		
			mean	median	
**First Symptom recognition to First health care visit**					**%<3 months**
	Benbakhta 2015 [15]	Morocco	3,2		
	Elzawawy 2008 [22]	Egypt	1		77
	Ermiah 2012 [23]	Libya		4	45,5
	Ibrahim 2012 [24]	Nigeria	12,2		18,4
	Moodley 2016 [11]	South Africa	5,5		
	Moodley 2018 [16]	South Africa		0,8	40,0
	Mousa 2011 [25]	Egypt	6,2	2,3	57
	Odongo 2015 [26]	Uganda	22,6	13	11
	Olajdje 2014 [27]	Nigeria	17		
	Pace 2015 [28]	Rwuanda		5	
	This study	Mali	13,4	4,8	42,6
					**%<1 month**
**First health care visit to Diagnosis**	Benbakhta 2015 [15]	Morocco	1,1		
	Ermiah 2012 [23]	Libya			84,5
	Moodley 2016 [11]	South Africa	3		
	Moodley 2018 [16]	South Africa		0,9	
	Mousa 2011 [25]	Egypt	1,7	0,6	
	Pace 2015 [28]	Rwuanda		5	
	This study	Mali	6,4	0,9	50
					**%<1 month**
**Diagnosis to Treatment**	Benbakhta 2015 [15]	Morocco	1,1		
	Dedey 2016 [29]	Ghana	1,1		46,1
	Moodley 2018 [16]	South Africa		1,2	
	This study	Mali	2,5	1,3	40,7

A problem in comparing the results is the reporting of study results: some studies only reported mean or median times and some only the proportions of women with a certain time delay. Independent of the reporting style, large time differences between countries and within countries for all time intervals, but especially for the interval between first symptom recognition and first healthcare visit, were reported. These differences probably reflect differences in the study population and study setting, but might also be explained by the different methodologies used to collect time points, such as the use of medical records, differences in interview technique, reporting biases and definitions of time points. To make study results more comparable in the future, the Aarhus statement to improve the design and reporting of studies on early cancer diagnosis should be considered [20].

This study shows that the median time length to first healthcare visit is almost five times as long as the consecutive time to diagnosis and this data supports the results from comparable studies focusing on early breast cancer diagnosis [7, 8], which found that awareness and knowledge about breast cancer and breast self-examination trigger recognition of initial breast cancer symptoms and timely healthcare seeking. The positive association of advanced diseases (T3/T4 tumours) with time to first healthcare visit [28] and shorter survival times [5] was further supported in our study. These results generally led to the conclusion that population-wide awareness and education programs are needed to improve the recognition of symptoms and promote prompt healthcare seeking to improve early diagnosis and survival [2]. Use of traditional medicine was another factor associated with prolonged time to first healthcare visit in our and comparable studies from sub-Saharan Africa [7, 8]. We also found shorter overall survival times for women visiting traditional healers before the first healthcare visit. Use of traditional medicine might have a negative impact on survival when it leads to advanced stages of the disease and when it is used as an alternative to standard cancer therapy. Cooperation with traditional healers and their education about symptoms have been suggested to improve early breast cancer diagnoses [30]. However, qualitative studies found that patients use traditional medicine during the whole patient journey, from first symptom recognition to treatment with a complementary or alternative treatment [10], and that reasons for using traditional medicine are manifold and also related to a lack of access and availability or low quality of healthcare services or it can be due to financial barriers, as also reported in other health contexts in Mali [31, 32]. Having no health insurance increased the time to first healthcare visit and reduced the survival time in our study. Health insurance was often not considered in comparable studies. In Mali as in most countries in sub-Saharan Africa, only a minority of the population has any health insurance coverage. We do not know if the better general socioeconomic status of health insured patients might have an impact on survival or if this is related to reduced patients’ out-of-pocket costs and the possibility of adhering to the treatment plan or seeking better treatment. Nevertheless, as costs for breast cancer treatment exceed the average income of families in sub-Saharan Africa [33], universal health coverage that includes costs for breast cancer care might have a great impact on survival, if adequate treatment options are available.

Half of the patients had a diagnostic service performed at the pathology department within 1 month, but 25% took 6 months and longer. Older women seem to receive a diagnosis faster after a first healthcare visit, probably because medical doctors associate cancer more with older women and, among younger women, breast related symptoms might often fall in times of breast feeding and be difficult to differentiate from breast cancer symptoms [34]. Working women, which includes all women having another status than being a housewife, took longer to receive a diagnosis and to begin treatment, which was probably due to time constraints and the need to support the family financially. The longer time to treatment start might also reflect more extensive and time-consuming diagnostic investigations and could also explain the increased time to beginning treatment for women with health insurance. Women who first visited a community healthcare center or a generalist took longer to receive a diagnosis, probably due to limited inward diagnostic facilities and the need to refer patients for further investigations. Similar results were reported in a study in Egypt [25] and can also reflect shortcoming in diagnosis and referral at this level of the healthcare system, for example, women who were told they had an infection or nothing at their first healthcare visit took longer to receive a diagnosis. Education programs for medical doctors or nurses working at the entry level of the healthcare system to enable them to recognize and communicate breast cancer symptoms and to provide referral might be useful to improve early diagnosis [2], but since other studies showed that low social support, lack of money, fear of diagnosis and mistrust in modern medicine are also barriers for early diagnosis [10, 11], such programs can only be part of larger health system strengthening and education programs.

Married women took longer to obtain a diagnosis and begin treatment in our study, which might reflect their dependency on their husband to make important decisions about healthcare and the sometimes low or negative support from husbands as reported elsewhere [10, 32].

An unexpected finding was that women living outside Bamako received a diagnosis faster than women living in Bamako, since the distance to the pathology service is longer, which was shown to be a barrier for early stage diagnosis of breast cancer in patients [35]. Whether this result is random or describes systematic differences for women from Bamako and outside the area needs to be investigated in future studies. Seeing that patients from Bamako began treatment earlier, was an expected finding since treatment facilities are close by and transport and accommodation of the accompanying family is cheaper and easier to organize. Shorter times to treatment were also found for women with larger tumour size, probably because of the greater urgency for treatment perceived by the medical doctors and especially the patient and her family.

While we found several factors associated with the lengths of the different time intervals and survival, we could also show that at least 16%, but probably up to 28% (assuming that the lost to follow-up cases did not start any treatment), of all patients did not start any treatment. This result highlights the bottleneck of early detection efforts. We further found that at least 37%, but probably up to 47%, of all patients died within 18 months after receiving a diagnosis. A study from Nigeria that followed-up breast cancer patients between 1 week and 6 years reported that 90% of all patients (from between 39% and 87%, including all patients lost to follow-up) who died, did so within the first year after diagnosis [36]. This high mortality rates are probably related to the advanced disease at time of diagnosis and lack of access, availability and affordability of treatment options in the countries.

### Limitations

This study followed-up all patients for at least 18 months that had received a diagnosis of breast cancer at the only pathology department in Mali during the first four months of 2016. A considerable effort was made to obtain precise time points for first symptom recognition, first healthcare visit and beginning of treatment and death (use of calendar techniques, verification by medical records provided by the patients and by searching medical records in the two main hospitals for cancer treatment in Bamako). Still, we lost ten patients during the follow-up time, who very likely did not receive any treatment and have probably died. A weakness of the study is the relatively small number of patients that were included in the study, therefore multivariable analyses on factors leading to delays were not conducted. As with other patient studies, this study does not include breast cancer patients who did not arrive at the hospital for diagnostic services and therefore important barriers for first healthcare seeking and diagnosis might be missing. It is also unknown how many women get lost after a first healthcare visit or never seek healthcare and these proportions might also vary largely between studies. These limitations effect the generalizability of our results.

### Implications

The results show that a continuum of breast cancer care is currently not given for the majority of patients in Mali, but has to be considered to fully benefit from any early detection programs and to improve survival of breast cancer patients. Focus should be on financial barriers at the patient level but also on barriers in providing adequate diagnostic and treatment services, including the needed medical work force. Only if the healthcare system can warrant a continuum of care for the majority of diagnosed breast cancer patients, would breast cancer awareness campaigns with down-staging strategies be useful. International efforts in providing standard diagnostic and treatment options, palliative care at low cost and training for medical doctors should be prioritised to improve breast cancer survival in Mali and other sub-Saharan countries.

### Future research

Prospective studies that accompany breast cancer patients from diagnosis to treatment outcome, analysing barriers to start of treatment, treatment adherence and clinical and patient reported outcomes are needed to describe in detail the situation of diagnosed breast cancer patients in sub-Saharan Africa and to highlight where public health interventions are needed most. The impact of the disease on the socioeconomic situation of the patient and her family needs to be studied and should be more the focus when discussing strategies to improve breast cancer early detection, treatment and survival in sub-Saharan Africa. Studies focusing on the macro level, for example, international relations, trade and laws, could be helpful to highlight barriers in providing care for breast cancer patients in sub-Saharan countries.

## Supporting information

S1 Questionnaire Original study questionnaire in French. (PDF)

S2 Questionnaire Study questionnaire translated into English. (PDF)

S1 Dataset Original anonymized dataset of the study. (SAV)
