# Supplementary material for: Factors associated with time to first healthcare visit, diagnosis and treatment, and their impact on survival among breast cancer patients in Mali
Source: PLoS One. 2018 Nov 29;13(11):e0207928. doi: 10.1371/journal.pone.0207928 (PMC6264812; doi:10.1371/journal.pone.0207928)
Supplement: S1 Questionnaire — (PDF) [file pone.0207928.s001.pdf]

Module 1 : Socioéconomique

1.1 N° fiche d'enquête : ______________/

1.2. N° du dossier : __________________/

1.3. Nom et Prénoms : ____/

1.4 Avez-vous une assurance médicale ?________/
1= Oui 2= Non 3= Autres_________________/

1.5 Qui a assuré le financement de votre diagnostic et traitement ? _____/______/______/
1= Vous-même 2= Votre mari/famille 3= Autres__________________________/

1.6 Avant votre maladie du sein, avez-vous eu la connaissance de l´autopalpation ?________/
1= Oui 2= Non 3= Autres____/

Module 2 : Première changement du sein, avant la première consultation

2.1 Quel était le(s) premier(s) changement(s) dans votre sein(s) ?_____/_____/___________/
1= Nodule ou tuméfaction 2= Douleur 3= Autres_______________________________/

2.2 Quand vous avez perçu de(s) premier(s) changement(s) dans votre sein(s) ?

***Date la plus précise possible (Calendrier) :**______________________*(jj/mm/aaaa)*

2.3. Estimation de la date:________/
1=Date approximative 2=Date précise

2.4 Quelle a été votre première pensée par rapport au changement(s) ?__________________/
1= Rien de grave 2= Une infection 3= Un cancer 4= Autres ___________________/

2.5 Qu´est-ce que vous a empêcher de pas consulté un médecin?__________/
1= Rien 2=Manque de la connaissance des symptômes 3= Manque de l´argent
4= Autres ____/

2.6 Avez-vous vu un tradi- thérapeute avant de consulter un médecin?_____________/
1= Oui 2= Non

Module 3 : Première consultation d´un médecin

3.1 Quand vous avez consulté la première fois un médecin ?

***Date la plus précise possible (Calendrier) :**___________________*(jj/mm/aaaa)*

3.2 Estimation de la date:________/
1=Date approximative 2=Date précise 3=Date vérifiée par_______________

3.3 Qu´elle était la raison qui vous a poussé à consulter un médecin? ________/
1= Connaissance des signes de cancer 2= Persistance/aggravation du changement
3= Recommandation d´une autre personne 4= Autres________________________________/

3.4 C'était quel type de médecin ?_________/
1= Généraliste 2= Gynécologue 3= Oncologue 4= Chirurgien 5= Radiologue
6= Autres____/

3.5 Le médecin a travaillé dans quelle structure de santé sanitaire ?____________/
1= CS Com 2= CS Ref 3= Hôpital 4= Clinique privé 5=Autres_______________ /

3.6 Qu´est-ce que ce médecin vous a dit par rapport à votre changement du sein ?_____/____/
1=Rien du grave 2=Une inflammation 3= Qqc sérieux 4=Peut-être un cancer
5=Rien 6= Autre____/

3.7 Qu´est-ce que ce médecin vous a recommandé ?_______/______/______/______/______/
1= Mammographie 2= Echographie 3=Biopsie 4=Cytoponction
5= Chirurgie 6= Traitement Médical 7=Rien 8= Autres________/

3.8 Est-ce que ce médecin vous a référé?________________/
1=Oui 2=Non 3= Autres________________________________/

3.9 Qu'est-ce que vous avez fait après cette première consultation ?_____/_____/______/
1= Obéir aux conseils du médecin 2= Consulté un autre médecin
3= Vu un tradi- thérapeute 4=Autres____________________________________/

Module 4 : Après la première consultation jusqu´au diagnostic

4.1 Combien des consultations (compter aussi plusieurs des consultations chez le même médecin) vous avez fait pour avoir le diagnostic *? (Inclus la première consultation et le diagnostic*). _________/

Apres votre première consultation et avant le diagnostic vous avez consulté :

	Type de médecin (a)	*Structure sanitaire (b)*	*Diagnostic (d)*	*Conseille (e)*	*Date référé chez Point G*
4.2					
4.3					
4.4					
4.5					
4.7					
4.8					
4.9					
	1= Généraliste<br>2=Gynécologue<br>3=Oncologue<br>4=Chirurgien<br>5= Autres	*1=CS Com<br>2= CS Ref<br>3= Hôpital<br>4= Clinique privé<br>5=Autres*	*1= Rien du grave<br>2= Inflammation<br>3= Qqc sérieux<br>4= Cancer<br>5= Rien<br>6=Autres*	*1= Mammographie<br>2= Echographie<br>3=Biopsie<br>4=Cytoponction<br>5= Chirurgie<br>6=Traitement Médical<br>7=Rien<br>8=Autres*	

Module 5 : Le diagnostic

5.1 Quand est-ce le diagnostic de votre cancer a été posé?

Date la plus précise possible (Calendrier) :___________________(jj/mm/aaaa)*

5.2 Estimation de la date :________/
1=Date approximative 2=Date précise 3= Date vérifiée par______________/

5.3 C'était quel type de médecin qui a fait le diagnostic?___________________________/
1= Généraliste 2=Gynécologue 3=Oncologue 4=Chirurgien 5= Radiologue
6= Le dernière médecin qui vous avez consulté, continuer avec 5.5 7=Autres___________ /

5.4 Le médecin qui a fait le diagnostic a travaillé dans quel type structure sanitaire?_______/
1=CS Com 2=CS Ref 3= Hôpital 4=Clinique privé 5=Autres_______________ /

5.5 Sur quelle base le médecin a fait le diagnostic?_____/_____/_____/_____/
1= Mammographie 2= Echographie 3=Biopsie 4=Cytoponction
5= Autres____/

5.6 Si vous avez fait une biopsie/ cytoponction, quand vous avez reçu le résultat?

Date la plus précise possible (Calendrier) :___________________(jj/mm/aaaa)*

5.7 Estimation de la date :________/
1=Date approximative 2=Date précise 3= Date vérifiée par______________/

5.8 Combiens des investigations avez-vous fait avant d´avoir le diagnostic?
a) Mammographie ___________/ b) Echographie ___________/ c) Biopsie __________/
d) Cytoponction____________/ e) Chirurgie___________/

5.9 Le médecin a conseillé quel(s) type(s) de(s) traitement(s) ?______/_______/_______/
1= La chirurgie 2= La chimiothérapie 3= La radiothérapie 4=Autres ____________/

5.10 Juste après, quelle a été votre opinion sur le traitement ?_________/
1=Vous voulez le faire le plus vite possible 2=Vous avez hésité de le faire
3=Vous ne voulez pas le faire 4=Autres________________________ /

5.11 Quelles difficultés avez-vous (eu) avant de commencer votre traitement?____/____/___/
1= Pas de difficulté 2=Avoir un rendez-vous 3= Manques d´argents
4= Manques des médicaments 5= Peur
6=Autres____/

5.12 Avez-vous déjà commencé votre traitement ? ___________________/
1=Oui 2= Non (continuer avec Module 7.)

Module 6 : Le traitement

6.1 Dans quelle structure sanitaire avez-vous commencé votre traitement ? ______________/
1=CS Ref 2= Hôpital 3=Clinique privé 4= Autre ______________________/

6.2 Avec quelle type de traitement avez-vous commencé________________/
1= La chirurgie 2= La chimiothérapie 3=Autre_________________________________/

6.3 Quand vous avez commencé/ fait le traitement ?

Date la plus précise possible (Calendrier) :____________________(jj/mm/aaaa)*

6.4 Estimation de la date :________/
1=Date approximative 2=Date précise 3= Date vérifiée par

6.5 Avez-vous déjà continué avec un deuxième traitement ? ______________________/
1=Oui 2= Non (continuer avec Module 7)

6.6 Avec quelle type de traitement avez-vous continué________________/
1= La chirurgie 2= La chimiothérapie 3=Autres___________________________/

6.7 Dans quelle structure sanitaire avez-vous continué votre traitement ? _________/
1=CS Ref 2= Hôpital 3=Clinique privé 4=Autres _______________/

6.8 Quand vous avez continué le traitement ?

Date la plus précise possible (Calendrier) :_______________(jj/mm/aaaa)*

6.9 Estimation du date :________/
1=Date approximative 2=Date précise 3= Date vérifiée par _____________/

Module 7 : Suivie

8.1. Date du dernier contact : _____________*(jj/mm/aaaa)*

8.2. La patiente est décédée le : _____________*(jj/mm/aaaa)*

Merci pour votre support !
