# Supplementary material for: Factors associated with time to first healthcare visit, diagnosis and treatment, and their impact on survival among breast cancer patients in Mali
Source: PLoS One. 2018 Nov 29;13(11):e0207928. doi: 10.1371/journal.pone.0207928 (PMC6264812; doi:10.1371/journal.pone.0207928)
Supplement: S2 Questionnaire — (PDF) [file pone.0207928.s002.pdf]

Module 1 : Patient information

1.1 N° medical record: ______________/

1.2. N° du dossier: __________________/

1.3. Name: ____/

1.4 Do you have a health insurance?________/
1= Yes 2= No 3= Other_________________/

1.5 Who pays for your diagnosis and treatment? _____/______/______/
1= You 2= Your husband/family 3= Other___________________________/

1.6 Before your illness, did you perform breast-self-examination?________/
1= Yes 2= No 3= Other____/

Module 2: First breast changes and first health care visit

2.1 Which was/were the first change(s) of your breast?_____/_____/___________/
1= Breast lump 2= Pain 3= Other_________________________________/

2.2 When did you recognise the first change(s) of your breast

Date the most precise (Calendar) :______________________(dd/mm/yyyy)*

2.3. Estimation of the date:________/
1=Date approximate 2=Date precise

2.4 What was your first interpretation of the symptoms?__________________/
1= Nothing serious 2= Infection 3= Cancer 4= Other ___________________/

2.5 What hindered you from seeking health care immediately?______________________
1= Nothing 2=No knowledge about the symptoms 3= Lack of money
4= Other ____/

2.6 Did you visit a traditional healer before seeking health care?_____________/
1= Yes 2= No

Module 3 : First health care visit

3.1 When did you visit a health care provider the first time ?

Date the most precise (Calendar) :____________________(dd/mm/yyyy)*

3.2 Estimation of the date:________/
1=Date approximate 2=Date precise 3=Date verified by_______________

3.3 What was the reason that made you visit the health care provider? ________/
1= Knowledge of breast cancer symptoms 2= Persistence/aggravation of the symptom
3= Recommendation of another person 4=Other________________________________/

3.4 Type of medical doctor you visited?_________/
1= Generalist 2= Gynaecologist 3= Oncologist 4= Surgeon 5= Radiologist
6=Other____/

3.5 The doctor was working in which structure of the health care system?____________/
1= Community Health Care Centre 2= Referral Hospital 3= Hospital 4= Private Clinic
5=Other _______________ /

3.6 What did the medical doctor tell you regarding your breast related symptoms?_____/____/
1=Nothing serious 2=Infection 3= Something serious 4=May be a cancer
5=Nothing 6= Other____/

3.7 What did the medical doctor recommend?_______/______/______/______/______/
1= Mammography 2= Ultrasound 3=Biopsy 4=Fine needle aspiration
5= Surgery 6= Medical Treatment 7=Nothing 8= Other________/

3.8 Did the medical doctor refer you?________________/
1=Yes 2=No 3= Other________________________________/

3.9 What did you do after that first consultation?_____/_____/______/
1= Followed the advice 2= Visited another medical doctor
3= Visited a traditional healer 4=Other_____________________________________/

<u>Module 4 : After the first health care visit up to diagnosis</u>

4.1 How many consultation did you have to receive a diagnosis (count also repeatedly consultations at the the medical doctor)*<u>? (Include the first consultation and the one to receive the diagnosis</u>*). _________/

After the first consultation and before the diagnosis you visited: :

	Type of medical doctor (a)	*Structure in the health system (b)*	*Diagnosis (d)*	*Recommendation (e)*	*Date referred to Point G*
4.2					
4.3					
4.4					
4.5					
4.7					
4.8					
4.9					
	1= Generalist<br>2=Gynaecologist<br>3=Oncologist<br>4=Surgeon<br>5=Other	*1=CHC<br>2= Referral Hospital<br>3= Hospital<br>4= Private Clinic<br>5=Other*	*1= Nothing serious<br>2= Inflammation<br>3= Sth. serious<br>4= Cancer<br>5= Nothing<br>6=Other*	*1= Mammography<br>2= Ultrasound<br>3=Biopsy<br>4=Fine needle aspiration<br>5= Surgery<br>6=Medical treatment<br>7=Nothing<br>8=Other*	

Module 5 : Diagnosis

5.1 When did you receive the diagnosis?

***Date the most precise (Calendar) :____________________**_(dd/mm/yyyy)_

5.2 Estimation of the date:________/
1=Date approximate 2=Date precise 3= Date verified by_______________/

5.3 Which type of medical doctor made the diagnosis?______________________________/
1= Generalist 2=Gynaecologist 3=Oncologist 4=Surgeon 5= Radiologist
6= The last one you visited continue with 5.5 7=Other___________ /

5.4 In which structure of the health care system did you receive the diagnosis?_______/
1=CHC 2=Referral Hospital 3= Hospital 4=Private Clinic
5=Other_______________ /

5.5 What was the base of the diagnosis?_____/_____/_____/_____/
1= Mammography 2= Ultrasound 3=Biopsy 4=Fine needle aspiration 5=Other____/

5.6 When you had a biopsy/fine needle aspiration when did you receive the result?

***Date the most precise (Calendar) :____________________**_(dd/mm/yyyy)_

5.7 Estimation of the date:________/
1=Date approximate 2=Date precise 3= Date verified by_________/

5.8 How many diagnostic services did you have before receiving a diagnosis?
a) Mammography ___________/ b) Ultrasound ___________/ c) Biopsy __________/
d) Finde needle aspiration____________/ e) Surgery___________/

5.9 The medical doctor recommended which type(s) of treatment?______/_______/_______/
1= Surgery 2= La chemotherapy 3= La radiotherapy 4=Other____________/

5.10 Immediately after that, what was your reaction?_________/
1=You wanted to have treatment as soon as possible 2=You hesitated to start treatment
3=You did not want to start treatment 4=Other __________________ /

5.11 Which difficulties did you have to start your treatment?____/____/___/
1= No difficulties 2=To have an appointment 3= Lack of money
4=Unavailability of drugs 5= Anxiety 6=Other____________/

5.12 Did you already stat your treatment? ____________________/
1=Yes 2= No

Module 6 : Treatment

6.1 In which structure of the health care system did you start the treatment? ___________/
1=Referral Hospital 2= Hospital 3=Private Clinic 4= Other____________/

6.2 With which kind of treatment did you start________________/
1= Surgery 2= Chemotherapy 3=Other________________________________/

6.3 When did you start the treatment?

Date the most precise (Calendar) :____________________(dd/mm/yyyy)*

6.4 Estimation of the date:________/
1=Date approximate 2=Date precise 3= Date verified by___________/

6.5 Did you already started a second treatment ? ______________________/
1=Yes 2= No

6.6 In which structure of the health care system did you start the next treatment? _________/
1=Referral Hospital 2= Hospital 3=Private Clinic 4= Other____________/

6.7 With which kind of treatment did you continue________________/
1= Surgery 2= Chemotherapy 3=Other________________________________/

6.8 When did you start the treatment?

Date the most precise (Calendar) :____________________(dd/mm/yyyy)*

6.9 Estimation of the date:________/
1=Date approximate 2=Date precise 3= Date verified by___________/

Module 7: Survival

8.1. Date of last contact: _____________*(dd/mm/yyyy)*

8.2. Date the patient died: _____________*(dd/mm/yyyy)*

Thanks for your support !
